# The role of RNA-binding protein, microRNA and alternative splicing in seed germination: a field need to be discovered

**DOI:** 10.1186/s12870-021-02966-y

**Published:** 2021-04-21

**Authors:** Xiaofei Xue, Fuchao Jiao, Haicheng Xu, Qiqing Jiao, Xin Zhang, Yong Zhang, Shangyi Du, Menghan Xi, Aiguo Wang, Jingtang Chen, Ming Wang

**Affiliations:** 1 College of Agronomy, Qingdao Agricultural University, Qingdao, 266109 China; 2 Dryland-Technology Key Laboratory of Shandong Province, Qingdao Agricultural, Qingdao, 266109 China; 3 Administrative Committee of Yellow River Delta Agri-High-Tech Industry Demonstration Zone, Dongying, 257347 China; 4 Shandong Institute of Pomology, Tai’an, 271000 China; 5 Jinan Fruit Research Institute, All China Federation of Supply and Marketing Co-operatives, Jinan, 250000 China; 6 Shandong Academy of Agricultural Sciences, Jinan, 250000 China

**Keywords:** Seed germination, RNA-binding protein, microRNA, Alternative splicing

## Abstract

Seed germination is the process through which a quiescent organ reactivates its metabolism culminating with the resumption cell divisions. It is usually the growth of a plant contained within a seed and results in the formation of a seedling. Post-transcriptional regulation plays an important role in gene expression. In cells, post-transcriptional regulation is mediated by many factors, such as RNA-binding proteins, microRNAs, and the spliceosome. This review provides an overview of the relationship between seed germination and post-transcriptional regulation. It addresses the relationship between seed germination and RNA-binding proteins, microRNAs and alternative splicing. This presentation of the current state of the knowledge will promote new investigations into the relevance of the interactions between seed germination and post-transcriptional regulation in plants.

## Background

A seed is a grain or ripened ovule of a plant that is used for sowing, and it is considered a condensed form of the plant. Under suitable environmental conditions, it can germinate from a physiological quiescence status, mobilize its reserves, biosynthesize new proteins, regenerate organelles, and produce cell membranes, eventually protruding the radicle and entering seedling establishment. Seed germination refers to a series of physiological and morphogenetic processes, which activates many new biological processes, including DNA replication, enzyme activation, cell division, membrane and mitochondrial repair, protein synthesis, generation of a sufficient energy supply, seedling growth [1, 2]. In dicotyledons, the embryonic root first emerges from the seed. It can make the seedling anchor in the ground. After water absorbing, an embryonic shoot emerges from the seed [3, 4]. In monocotyledons seeds, the embryo’s cotyledon and radicle are covered by a coleoptile and coleorhiza, respectively. The coleorhiza grows out of the seed first, followed by the radicle. The coleoptile is then pushed up until it reaches the surface [3, 4]. The regulatory network of seed germination is mediated by many factors, e.g., temperature, water, oxygen, light, nutrition, phytohormones, and mRNA oxidation [5–8].

Post-transcriptional regulation refers to the regulation of gene expression at post-transcriptional level. It plays an important role in regulating gene expression [9–11]. The expression of the different genes is post-transcriptionally regulated by many factors, such as RNA-binding proteins (RBPs), microRNAs (miRNAs), spliceosome [12–15]. RNA-binding proteins are proteins that act as important regulators of many processes such as alternative splicing, mRNA nuclear export, mRNA stability, and translation. RNA-binding protein can achieve these events through an RNA recognition motif (RRM) that allows binding of the RNA-binding protein to a secondary structure or specific sequence in their target transcripts. RNA-binding proteins play a pivotal role in post-transcriptional regulation of different RNAs, such as splicing, mRNA stability, polyadenylation, mRNA localization [11, 16, 17]. microRNAs (miRNAs) also participate in the post-transcriptional regulation of gene expression. miRNA is a type of endogenous small non-coding RNA molecule with a length of about 20–24 nucleotides, which has many important regulatory functions in cells. Each miRNA can have multiple target genes, and even several miRNAs can also pair with the same target gene [14, 18]. miRNAs can influence the translation of the mRNA, drive mRNA cleavage or shorten the poly(A) tail [19, 20]. Alternative splicing is mediated by the spliceosome. The spliceosome is a kind of large and complex molecular machine within the nucleus of cells [21]. It mainly participates in mRNA processing by removing introns from a transcribed pre-mRNA [22]. Splicing can also generate the premature stop codons that recruit the NMD (nonsense mediated decay) machinery [21, 23]. The proteins translated from alternatively spliced mRNAs would have different amino acid sequence. Consequently, the protein structure and biological functions would be changed. Post-transcriptional regulation is also reflected in the splicing and processing of the mRNA precursor hnRNA, the process and positioning of the mRNA, the stability of the mRNA and its degradation, RNA editing.

Understanding the seed germination regulatory network can help us to better understand the situation of the seed itself and improve the germination rate of the seed and the development status of the seedling. This review addresses the relationship between seed germination and post-transcriptional gene regulation in plants. It provides insight into the role of some important mechanisms of post-transcriptional regulation: RNA-binding protein, miRNA and alternative splicing. We also raise some questions about future studies in the discussion section.

### Seed germination and RNA-binding proteins

RNA-binding protein can bind to single stranded RNA or double stranded RNA in eukaryotic cells, and plays a key role in almost all aspects of post-transcriptional regulation [17, 24]. RNA-binding proteins are characterized by the presence of different RNA-binding domains, e.g. RNA recognition motif (RRM), K-homology (KH) domain, zinc finger structure, pentatricopeptide-repeat (PPR) domain [25–27]. In Arabidopsis, more than 200 different RNA-binding proteins were identified in the genome, and most of them were plant-specific, suggesting specific functions in plant physiology [24]. The relationship between RNA-binding proteins and seed germination has been examined in numerous studies. Narsai et al. (2011) carried out an in-depth transcriptomic profiling in Arabidopsis to investigate the regulatory mechanism of seed germination. More than 10,000 genes were differentially expressed during cold stratification, revealing an active period in preparing seeds for germination [28]. Among them, genes encoding proteins involved in RNA-binding functions, included RNA helicases, RNA-binding proteins, and ribonucleases, were identified in transcriptomic profiling. Further analysis showed that 137 PPR domain-containing genes that have been shown to have roles in RNA splicing, cleavage, editing, stability, and translation displayed transient expression during germination, and of these, 75 genes showed germination-specific expression [28, 29]. In addition, the presence of mitochondrial DNA replication factors and RNA-processing functions in this germination-specific subset represents the earliest events in organelle biogenesis, preceding any changes associated with energy metabolism. This result also suggested that the collaboration among RNA-binding proteins, the mitochondrial origin and retrograde signals may be crucial for successful seed germination [28].

In plants, abscisic acid (ABA) is an important negative regulator of seed germination [30]. In Arabidopsis, Jung et al. (2013) characterized an ABA-regulated RRM-containing RNA-binding protein designated ARP1 (ABA-REGULATED RNA-BINDING PROTEIN 1) [31]. The experiment showed that ARP1 transcription was inhibited by ABA. Interestingly, both overexpression and knockdown *arp1* mutants resulted in delayed seed germination under ABA, high salt, and dehydration stress conditions. The identical phenotypes of the *arp1*-overexpressing mutant and the *arp1* knockdown mutant indicated that tight regulation of the *ARP1* transcript is necessary for normal germination. This result indicated that ARP1 affects ABA-regulated seed germination of Arabidopsis through post-transcriptional regulation [31]. In addition, MCT1 (MEI2 C-TERMINAL RRM ONLY LIKE 1) is another ABA related RRM containing protein in Arabidopsis. The study from Gu et al. (2016) showed that the transcript level of MCT1 was markedly increased upon ABA treatment [32]. Further analysis of MCT1-overexpressing mutant and *mct1* knockdown mutants demonstrated that MCT1 inhibited seed germination under ABA treatment. Moreover, the transcript levels of *ABI3*, *ABI4*, and *ABI5*, three ABA signaling-related genes, were increased by MCT1 [32]. Previous research indicated that *ABI3*, *ABI4*, and *ABI5* play a negative role in seed germination in Arabidopsis [33–37]. These studies showed that ABA-upregulated MCT1 plays a negative role in seed germination under ABA by modulating the expression of ABA signaling-related genes. Besides, abiotic stresses act as important effectors of seed germination, and they regulate seed germination through ABA [38, 39]. Various abiotic stress-related RNA-binding proteins have been determined to perform crucial roles in post-transcriptional regulation of RNA metabolism during seed responses to abiotic stresses. Studies examining glycine-rich RNA-binding proteins have indicated an important role for these proteins during Arabidopsis germination under abiotic stresses [40].

**Fig. 1:**
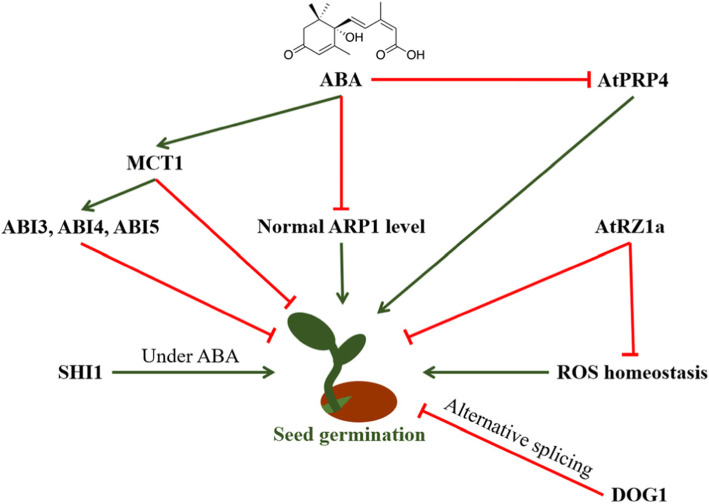
The relationship between seed germination and reported RNA-binding proteins. ABA influence the transcription of ARP1, whose level is important for seed germination. ABA also stimulates the transcription of MCT1, that inhibit seed germination directly or indirectly. Besides, ABA inhibits the the transcription of AtPRP4 which regulate seed germination positively. In addition, ROS homeostasis is also important for seed germination

GLYCINE RICH PROTEIN 2 (GRP2) is a glycine-rich RNA-binding protein. Fusaro et al. (2007) demonstrated that *AtGRP2* expression is regulated by cold in Arabidopsis [41]. Expression analysis revealed that the *AtGRP2* gene is active in meristematic tissues. Downregulation of the *AtGRP2* gene using gene-silencing techniques resulted in early flowering and an altered stamen number, and it affected seed development [41]. Further study indicated that AtGRP2 is a nucleocytoplasmic protein characterized by a nucleic acid-binding CSD domain, two glycine-rich domains and two CCHC zinc-fingers [42]. AtRZ-1a encodes a zinc finger-containing glycine-rich RNA-binding protein, and it has a negative impact on seed germination in Arabidopsis under salt or drought stress conditions [40]. Researchers showed that overexpressing the *atRZ-1a* mutant resulted in higher H_2_O_2_ levels and retarded germination compared with the wild type under salt or dehydration conditions. In contrast, the knockdown mutants of *atRZ-1a* germinated earlier under the same stress conditions. Moreover, germination of the transgenic plants and mutant lines was influenced by the addition of ABA. Furthermore, the research also showed that the transcription level of several genes, such as *ISO* (*isocitrate lyase*), *CRU* (*cruciferin*), *SSP* (*12S seed storage protein*) and *LEAP* (*LEA protein in group five*), were induced in the *atRZ-1a*-overexpressing mutant. Moreover, further proteome analysis identified the putative target genes of atRZ-1a, including ferredoxin-nitrate reductase, aconitate hydratase, glyceraldehyde 3-phosphate dehydrogenase, alcohol dehydrogenase, phosphoglycerate mutase, thylakoid lumenal protein, glutathione reductase, glutathione S-transferase, glutamate 1-semialdehyde aminomutase, carbonic anhydrase, and serine hydroxymethyl transferase [40]. This analysis revealed that the genes involved in reactive oxygen species homeostasis (important for seed germination, [43, 44]) were affected by atRZ-1a during seed germination (Fig. 1).

In addition, Park et al. (2017) investigated the functional roles of three zinc-finger RNA-binding proteins (RZs) in *Brassica rapa* [45]. The experiment showed that ABA can stimulate the expression levels of all three BrRZ proteins. During seed germination, the *BrRZ3*-overexpressing mutant showed retarded seed germination and stem growth under normal growth conditions, whereas it displayed decreased transcript levels of *β-glucosidase* (*BGU*) and *GA4 homology* (*GA4H*) and increased levels of *LEAP* and *SSP* in the presence of ABA. Germination of *BrRZ2-* or *BrRZ3*-overexpressing Arabidopsis seeds was delayed compared with that of wild-type seeds under dehydration, salt stress and cold stress conditions. Importantly, all BrRZs possessed RNA chaperone activity [45]. A previous report also indicated that RNA-binding proteins are involved in seed germination independent of ABA in Arabidopsis. SHINY1 (SHI1) is a K-homology domain-containing RNA-binding protein, and it interacts with CPL1 to regulate gene expression [46]. Jiang et al. (2013) reported that the *shi1* mutant is more sensitive to cold conditions during Arabidopsis vegetative growth and is insensitive to ABA during seed germination [46]. SHI1 can interact with SHI4/CPL1. Loss-of-function mutations in *shi1* and *shi4* resulted in similar changes in the expression patterns of some stress-related genes. Moreover, both *shi1* and *shi4* mutants display higher mRNA capping efficiency and altered polyadenylation site selection for some of the stress-related genes [46]. Except dicots, in recent years, a lot of proteomic studies conducted in monocot have been conducted in exploring the gene expression regulation, reserves mobilization and metabolisms reactivation, which brings us new insights on the mechanisms of metabolism regulation during this process. In *Oryza sativa*, proteomic analysis revealed the existence of KH domain containing protein and a glycine-rich RNA-binding protein is important for seed germination. Decreased glycine-rich RNA-binding protein level after absorbing water in seeds appear to be associated with seed germination [47]. Moreover, the glycine rich RNA-binding protein was down-regulated after seeds imbibition, which might promote germination. They supposed that the RNA-binding proteins probably involved in RNA stability by forming RNA chaperones during seed germination. In addition, the RNA-binding proteins play important role in keeping the stability and regulating the functions of the long-lived mRNAs, which was shown more than 17,000 stored mRNAs exist in dry rice seeds by previous study [48].

Collectively, these findings suggest a finely controlled but crucial role of RNA-binding proteins during germination. So far, how these activities are regulated in a coordinated and sequential manner is largely unknown. Moreover, other germination related RNA-binding proteins need to be discovered and studied in the future.

### Seed germination and miRNA

miRNAs are a small non-coding RNA molecule which participates in RNA silencing and post-transcriptional regulation of gene expression through mRNA cleavage or repressing translation [49–52]. In plants, miRNAs participate in a broad range of important seed related processes, including seed development, seed dormancy, seed germination [53]. By exploring the mechanism of seed germination, a variety of miRNAs which involved in phytohormone signaling responses, stress responses, antioxidant effect and regulation of key transcription factors have been identified, and are considered to play an important role in seed germination [54, 55]. Numerous miRNAs are present in the germinating seeds of Arabidopsis, some of which are involved in the regulation of germination by phytohormones, such as abscisic acid (ABA) and auxin. In plants, miRNAs can interact with ABA, an important germination regulator, to regulate seed germination. Reyes and Chua (2007) showed that in germinating Arabidopsis seeds, ABA induces the accumulation of mature miRNA159 in an *ABI3*-dependent way, and the miRNA159 mediates cleavage of the *MYB101* and *MYB33* transcripts (Table 1) [56]. These two MYB transcription factors function as positive regulators of ABA responses, and the knockdown mutants of *myb33* and *myb101* show hyposensitivity to ABA. Furthermore, the *miRNA159* over-expression mutant had reduced *MYB33* and *MYB101* transcript levels that rendered the plants hyposensitive to ABA. In addition, the accumulation of miRNA159 in the *abi3* mutant was reduced 2- to 3-fold with respect to that of wild type, indicating that *ABI3* regulates *miRNA159* expression [56]. Moreover, at higher ABA concentrations, the germination efficiency of the *miRNA159*-overexpressing mutant seeds was higher than the wild type seeds under the same conditions. This result suggested that the ABA-induced accumulation of miRNA159 is a homeostatic mechanism to induce *MYB33* and *MYB101* transcript degradation, which desensitizes the seed to ABA signaling during seed germination [56]. Besides, the functional roles of miRNA402 in Arabidopsis were also investigated under stress conditions [57]. In Arabidopsis, ABA was shown to regulate DNA demethylation through DNA glycosylase [60, 61]. Kim et al. (2010a) indicated that a putative DNA glycosylase, DEMETER-LIKE protein3 (DML3), which is involved in DNA demethylation, is the target of miRNA402 (Table 1) [57]. The expression of *DEMETER-LIKE protein3* mRNA was downregulated in *miRNA402*-overexpressing transgenic plants. The transcript level of *miRNA402* in the germinating seeds was significantly increased by dehydration, cold and salt, and the expression of the stress response markers *RD29A* or *RD29B* were significantly increased under these stresses. Moreover, 35S::miRNA402 seeds and *dml3* mutant seeds germinated earlier than wild-type seeds under salt stress conditions. This finding implies that mature miRNA402 plays a role as a positive regulator of seed germination of Arabidopsis under stress conditions, and that miRNA402-guided regulation of DNA demethylation is an adaptive process of plants to stress conditions [57].

**Table 1:** The type and sequence of seed germination related microRNA

miRNA	Sequence (5′-3′)	Target genes	References
miRNA159	UUUGGAUUGAAGGGAGCUCUA	MYB33, MYB101	[56]
miRNA402	UUCGAGGCCUAUUAAACCUCUG	DML3	[57]
miRNA160	UGCCUGGCUCCCUGUAUGCCA	ARF10, ARF16, ARF17	[58]
miRNA395c	CUGAAGUGUUUGGGGGGACUC	Unknown	[57]
miRNA395e	CUGAAGUGUUUGGGGGAACUC	APS, SULTR2;1	[57]
miRNA163	UUGAAGAGGACUUGGAACUUCGAU	PXMT1	[59]

In addition to ABA, auxin is also an important hormone that participates in seed germination. AUXIN RESPONSE FACTORS (ARFs) are transcription factors involved in auxin signal transduction [62]. By using derepression experiments wherein silent mutations are introduced into the ARF10 sequence complementary to miRNA160, Liu et al. (2007) showed that negative regulation of ARF10 by miRNA160 plays important roles in seed germination [58]. Transgenic plants expressing an miRNA160-resistant form of ARF10 exhibited developmental defects such as curled stems, serrated leaves, and contorted flowers. During germination, the transgenic mutant seeds were hypersensitive to ABA in a dose-dependent manner. In contrast, overexpression of *miRNA160* resulted in reduced sensitivity to ABA during germination. In line with that, Nonogaki (2008) also indicated that miRNA160 could influence seed germination by targeting ARF16 and ARF17 in Arabidopsis [63]. In addition, the expression of sulfate metabolism-related enzymes is also regulated by miRNAs during seed germination. Kim et al. (2010b) showed that the transgenic Arabidopsis *miRNA395c*-overexpressing mutant and the *miRNA395e*-overexpressing mutant have negative and positive effects on the seed germination of Arabidopsis, respectively, under salt or dehydration stress conditions [64]. This result indicated that germination of the *miRNA395c*-overexpressing mutant was significantly retarded compared with that of the wild type. In contrast, seed germination of the *miRNA395e*-overexpressing mutant was noticeably accelerated under salt or dehydration stress. Further analysis during seed germination showed that the mRNA cleavage of ATP sulfurylase (APS) and the sulfate transporter SULTR2;1 were mediated by the mature miRNA395e [64].

Recently, Chung et al. (2016) showed that light can regulate seed germination through influencing the expression of *miRNA163* [59]. In Arabidopsis, miRNA163 is 24 nucleotides in length, and it targets the transcripts encoding S-adenosyl-Met-dependent carboxyl methyltransferase (SAMT)-like proteins [59]. Chung et al. (2016) also showed that primary miRNA163 levels increased significantly under light. In contrast, the transcript levels of PXMT1, the target of miRNA163 that encodes a methyltransferase that methylates 1,7-paraxanthine, decreased under the same light conditions. Moreover, during seed germination, miRNA163 and its target PXMT1 are predominantly expressed in the radicle, and *mirna16*3 knockout mutant or PXMT1-overexpressing mutant showed delayed seed germination compared with the wild type under continuous light [59].

Various reports have also shown that miRNA and seed germination have complex regulatory networks in other plants. Huang et al. (2013) performed a comprehensive analysis of miRNAs during seed maturation in *Brassica napus* through using whole seeds and separate analyses [65]. Their study identified more than 500 conserved miRNA or variant unique sequences. The miRNA156 family was the most abundant in the seed followed by the miRNA159, miRNA172, miRNA167 and miRNA158 families. The functional classification revealed that their target genes were highly enriched in pathways of RNA, DNA, protein, S-assimilation, development, signaling and redox. Detailed inspection of the results revealed that some miRNAs function in the same pathway(s) by targeting different genes in the same family. In addition, they also predicted that miRNA173, miRNA400 and miRNA396 “cotarget” pentatricopeptide (PPR) repeat-containing proteins, which indicated an interaction between RNA-binding proteins and miRNA during seed germination. In addition, F-box family proteins are cotargets of miRNA156, miRNA394 and miRNA319.

Auxin response factors (ARFs) could be regulated by miRNA160, miRNA167, miRNA390 and miRNA156 coordinately [65]. In line with this, Jian et al. (2016) also suggested an important role of miRNA156 during seed germination of *Brassica napus* [66]*.* They constructed three small-RNA libraries from *Brassica napus* exposed to distilled water, drought, and salt during imbibition of the seed. The results indicated that under the distilled water, drought, and salt conditions, 85, 81, and 81 known miRNAs were found, respectively. miRNA156 showed the highest abundance, followed by miRNA167, miRNA166, and miRNA390, during the early stage of seed germination under salt and drought stresses [66]. Under drought conditions, miRNA156, miRNA169, miRNA860, miRNA399, miRNA171, and miRNA395 were significantly downregulated, whereas only miRNA172 was significantly upregulated. In contrast, only two miRNA families, miRNA393 and miRNA399, were significantly downregulated under salt stress. Moreover, drought-responsive family protein (DRRP), early responsive to dehydration stress protein (ERD), stress-responsive alpha-beta barrel domain protein (SRAP), and salt tolerance homolog2 (STH2) were confirmed as the targets of the identified miRNAs. Besides, the prediction of the identified miRNA showed that various transcription factors (including SBP, MYB, ARF, NAC, TCP, NF-YA, GRF) and metabolic process related proteins (such as F-box protein, ATP sulfurylase, CCHC-type zinc finger protein, NAD(P)-binding protein, ADP-ribose polymerase), were identified as conserved miRNA targets [66]. In *Nelumbo nucifera*, by using high-throughput small RNA sequencing, Hu et al. (2016) identified 145 known miRNAs and 78 novel miRNAs during seed germination. The largest miRNA family size identified was miRNA396, following by miRNA169 and miRNA393 possessed 12 and 8 members, respectively [67]. They also detected 2580 targets were detected for all the miRNAs by using degradome sequencing. Furthermore, both miRNA159b and miRNA319c targeted mRNA encoding the MYB transcription factor. miRNA156a, miRNA160a, miRNA160a-5p and miRNA169a targeted mRNA encoding many transcription factors, including SPL17, ARF18, NAC, NF-YA10. GO (Gene Ontology) and KEGG pathway analyses of the putative target genes showed that many target genes enriched in “regulation of transcription” and involved in “carbohydrate metabolism”, “amino acid and energy metabolism” [67].

In monocots, some studies also explored the regulatory mechanisms between miRNAs and seed germination. Kang et al. (2012) carried out a miRNA microarray analysis to study the expression of conserved miRNAs in maize seeds and identified 125 known miRNAs [68]. Further study indicated that miRNA319 had a significant influence on seed germination. Moreover, miRNA171 and miRNA166 also played important roles in seed germination. miRNA166 was predicted to target bZIP genes in maize [69], which could regulate many processes such as seed maturation and stress signaling [70]. miRNA171 was predicted to target the GRAS transcription factor that controlled gibberellic acid (GA) signaling and phytochrome A signal transduction in seeds [71]. He et al. (2015) carried out an experiment to explore the regulation mechanism of miRNA in the first 24 h after imbibition which is important for rice seed germination [72]. The analysis identified 289 miRNA loci, including 59 known and 230 novel miRNAs. Moreover, 17 conserved families were detected in the imbibed rice seeds, and miRNA319, miRNA168, miRNA156, miRNA166, and miRNA159 had high abundance after imbibition. However, some conserved miRNAs, i.e., miRNA164, miRNA395, and miRNA393, were lowly expressed in rice seeds. Besides, they also demonstrated that miRNA160, miRNA156/miRNA529, miRNA319 and miRNA159 can regulate seed germination by targeting the mRNAs coding ARFs, SPL, TCP, MYB transcription factors respectively [72].

### Alternative splicing

Alternative splicing is a finely regulated process during gene expression that leads to a single gene coding for multiple proteins. The interaction between seed germination and alternative splicing has been originally studied in Arabidopsis. A genomic study on ABA-regulated gene expression was performed to identify 1354 ABA-responsive genes and novel ABA signaling components [73, 74]. To further determine whether the splicing-related genes that encode small nuclear ribonucleoprotein particle (snRNP) proteins are regulated by ABA, Raab and Hoth (2007) analyzed the transcript abundances of genes that encode snRNPs using the ASRG database [75]. Their results suggested that an ABA-dependent repression was found for 25% of the 91 snRNP genes that indicated changed splicing activity in the presence of ABA. Moreover, the U4/U6-specific snRNP gene *AtPRP4* was repressed by ABA. The knockout of *AtPRP4* displayed retarded seed germination, suggesting that alternative splicing is crucial for Arabidopsis seed germination [75]. In recent years, increasing numbers of studies have investigated alternative gene splicing during seed germination. The Arabidopsis protein DOG1 (DELAY OF GERMINATION 1) is an important effector of seed dormancy. It was originally identified through QTL and the DOG1 locus shows sequence variation in both the promoter and coding region among its accessions [76]. Moreover, the variation in *DOG1* expression contributes to variations in seed dormancy levels [76]. Nakabayashi et al. (2015) showed that the *DOG1* gene is alternatively spliced during breakage of seed dormancy [77]. In their study, they indicated that the *DOG1* gene produces five transcript variants encoding three protein isoforms. The seeds of *dog1* mutant only transcripts single *DOG1* variants, and do not accumulate DOG1 protein. Further analysis showed that expressing two or more DOG1 transcript variants can lead to late germination and high accumulation level of DOG1 protein [77]. Moreover, DOG1 protein can bind to itself, and different DOG1 variant contained Arabidopsis seeds show different seed dormancy level. This finding suggests that the accumulation of DOG1 protein requires the presence of multiple isoforms. Interestingly, their study also indicated that the single isoforms are functional but require the presence of additional isoforms to prevent protein degradation [77].

In addition, Zhang et al. (2016) conducted RNA-sequencing experiments to dissect alternative splicing events in barley seed germination and once again emphasized the important role of alternative splicing in seed germination [78]. Their results identified between 552 and 669 common alternative splicing transcripts in germinating barley embryos from four barley varieties. Alternative 3′ splicing (34–45%), intron retention (32–34%) and alternative 5′ splicing (16–21%) were three major alternative splicing events during seed germination [78]. The KEGG pathway analysis showed that alternative splicing transcripts were predominantly mapped onto ribosome, RNA transport, spliceosome, oxidative phosphorylation, mRNA surveillance, protein synthesis, glycolysis, carbon metabolism, spliceosome. They also showed that auxin efflux carrier and SnRK2 were alternatively spliced during germination [78]. Moreover, correlation analysis of gene expression supported that alternative splicing of hormone responsive transcripts could coexist with gene transcripts responsible for protein biosynthesis and sugar metabolism, which revealed an important role of alternative splicing in barley seed germination [78]. Recently, Tognacca et al. (2019) carried out an experiment to study the effect of alternative splicing on the expression of light related genes during Arabidopsis seed germination. They evaluated transcriptome-wide changes in stratified seeds irradiated with a pulse of red light or far-red light. They showed that the red light changed the expression of 20% of the transcriptome and modified the alternative splicing pattern of 226 genes associated with a strong enrichment in the GO categories of mRNA processing, RNA splicing, and mRNA metabolic processes [79]. Furthermore, the experiment confirmed that red light controls the expression of alternative splicing related factors (At-SR30, At-RS31a, At-RS31, and At-U2AF65A), a light-signaling component (At-PIF6), and a dormancy-related gene (At-DRM1). Therefore, based on those result, they conclude that red light triggers alternative splicing changes in some splicing factors, phytochrome B, light-signaling components, and dormancy/germination regulators [79].

## Conclusions

**Fig. 2:**
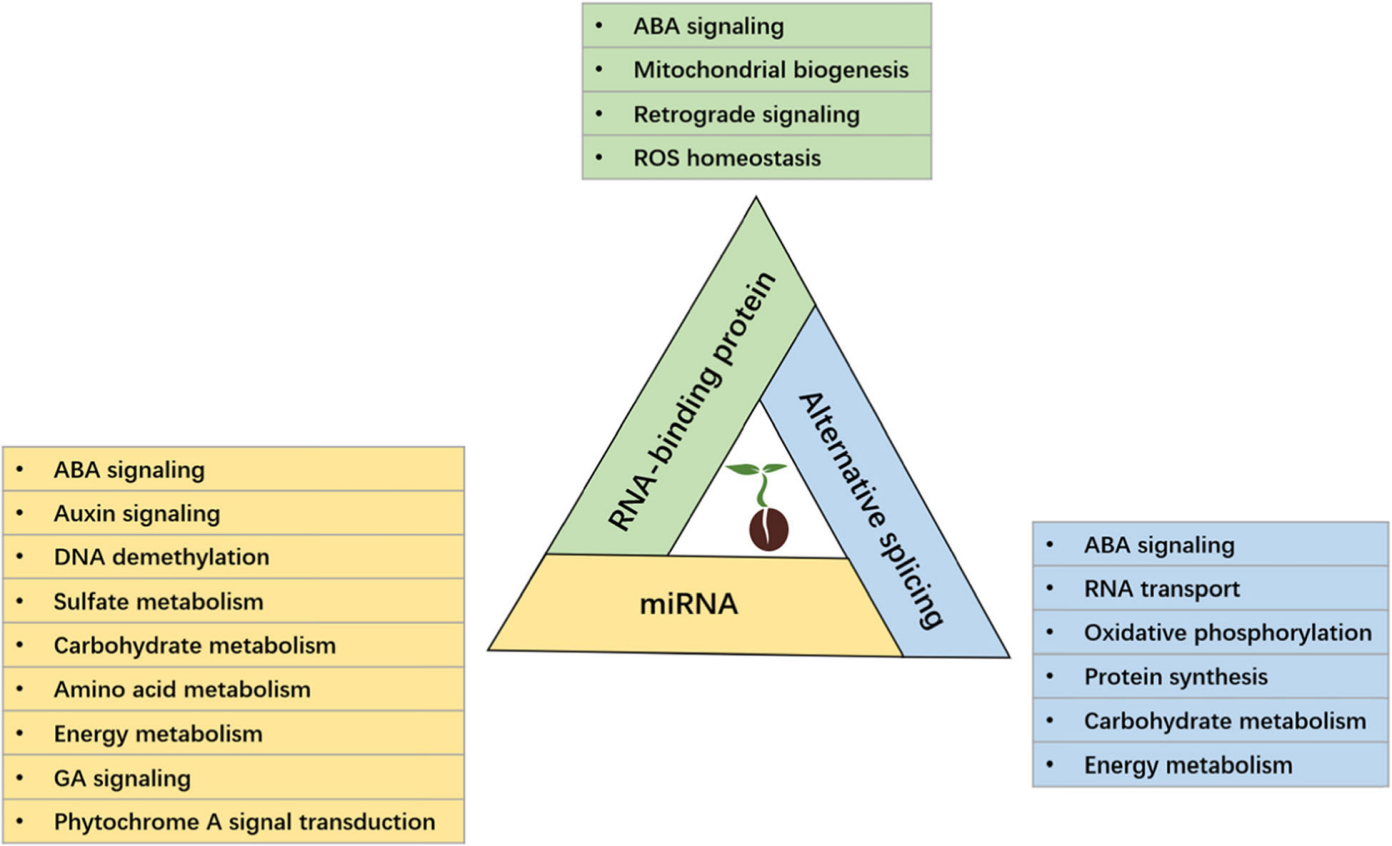
Various processes are regulated by RNA-binding proteins, miRNAs and alternative splicing during seed germination. There are also interactions between RNA-binding proteins, miRNAs and alternative splicing. miRNA biosynthesis is regulated by RNA-binding protein in plants [83, 84]. Alternative splicing can influence miRNA binding site [85]. Studies also showed that alternative splicing is mediated by RNA-binding proteins [86, 87]

Seed germination is a series of orderly physiological processes and morphogenetic processes from the beginning of seed swelling. It is also a complex process involving multiple levels of regulation [1, 4]. In plants, post-transcriptional regulation is an essential and important component of gene expression regulation. Numerous findings have unveiled and characterized various factors involved in post-transcriptional regulation, such as RNA-binding proteins, poly (A)-binding proteins (PABPs), miRNAs, lncRNAs, and snRNAs [9, 80–82]. Considering the tight and complex connection between post-transcriptional regulation and seed germination (Fig. 2), it will be interesting to discover the new mechanisms of post-transcriptional regulation in seed germination. Some recently developed technologies will assist with future studies. In addition, there are still many aspects of RNA decay that need to be studied deeply, such as the spliceosome and editosome (a large multiprotein complex that catalyzes RNA editing) that have a crucial role in post-transcriptional regulation. Thorough knowledge of the regulatory mechanisms of RNA-binding proteins and miRNAs during seed germination are scarce, taking into consideration their high numbers in plants. Recent studies have found that mRNA stability and oxidation are involved in the regulation of seed germination, and the completion of germination and the regulation of dormancy also depend on the degradation of specific subsets of mRNA, indicating a tight relationship at work between seed germination and post-transcriptional regulation [88–91]. In addition, post-translational regulation and protein modification also participates in the regulation of seed germination, such as mRNA decapping, protein phosphorylation, protein oxidation [92–95].

Here, we addressed many important connections between seed germination and post-transcriptional regulatory factors in plants, including RNA-binding proteins, miRNAs and alternative splicing. These findings may inspire us to investigate new regulatory mechanisms and to fill in the gaps in knowledge regarding the interactions between seed germination and post-transcriptional regulation in plants.

In conclusion, post-transcriptional regulation has attracted consideration in seed germination related processes. More work needs to be carried out to understand the functions and mechanisms related to the involvement of post-transcriptional regulation in germination-related processes. This kind of new knowledge could also pave the way for discovering a new and complex regulatory seed germination network in plants, and the new findings will provide new research directions for seed biology and enrich the field of seed germination.

## Data Availability

Data sharing is not applicable to this article as no datasets were generated or analysed during the current study.

## Abbreviations

RBPs: RNA-binding proteins
RRM: RNA recognition motif
NMD: nonsense mediated decay
KH: K-homology
PPR: pentatricopeptide-repeat
ARP1: ABA-REGULATED RNA-BINDING PROTEIN 1
MCT1: MEI2 C-TERMINAL RRM ONLY LIKE 1
GRP2: GLYCINE RICH PROTEIN 2
ISO: isocitrate lyase
CRU: cruciferin
SSP: 12S seed storage protein
LEAP: LEA protein in group five
BGU: β-glucosidase
GA4H: GA4 homology
SHI1: SHINY1
DML3: DEMETER-LIKE protein3
ARFs: AUXIN RESPONSE FACTORS
APS: ATP sulfurylase
DRRP: drought-responsive family protein
ERD: dehydration stress protein
SRAP: stress-responsive alpha-beta barrel domain protein
STH2: salt tolerance homolog2
DOG1: DELAY OF GERMINATION 1
